# BGC1201: A potent allosteric SHP2 inhibitor with broad antitumor activity in lung, pancreatic, and esophageal cancer xenografts

**DOI:** 10.1016/j.pscia.2026.100128

**Published:** 2026-06-15

**Authors:** Yuhuai Wang, Jiahui Xia, Haifeng Ding, Duxin Li

**Affiliations:** a College of Pharmaceutical Sciences, Soochow University, Suzhou, 215123, PR China; b BrightGene Bio-Medical Technology Co., Ltd., Suzhou, Jiangsu, 215123, PR China

**Keywords:** SHP2, BGC1201, Antitumor activity, Pharmacokinetics, p-ERK

## Abstract

BGC1201 is a novel allosteric SHP2 inhibitor developed via fragment-based drug design (FBDD). It potently inhibited SHP2-WT phosphatase with an IC_50_ of 2.47 nM and suppressed p-ERK expression in NCI-H358 cells with an IC_50_ of 17.3 nM. BGC1201 showed weak inhibition of the hERG potassium channel, with an IC_50_ value > 40 μM, suggesting a low hERG-related liability in vitro. In a panel of 14 tumor cell lines, BGC1201 showed antiproliferative activity against 12 lines, with IC_50_ values ranging from 0.0236 to 8.66 μM, while sparing normal HUVEC cells. In nude mouse xenograft models of human lung (NCI-H358), pancreatic (MIA PaCa-2), and esophageal (KYSE-520) cancers, oral administration of BGC1201 produced significant tumor growth inhibition with a dose-dependent trend. Western blot analysis further confirmed that BGC1201 reduced p-ERK levels in tumor tissues in a time-dependent manner. Pharmacokinetic evaluation in rats showed that BGC1201 achieved measurable oral exposure and was eliminated through multiple metabolic and excretory pathways. These findings support BGC1201 as a promising candidate for further anticancer drug development.

## 1 Introduction

With the continuous increase in the incidence of malignant tumors, cancer has become the leading cause of death worldwide [1]. Traditional chemotherapeutic drugs generally exhibit limited target selectivity, leading to systemic toxicities and drug resistance. Therefore, the development of novel targeted drugs has become an important direction in antitumor research.

Protein kinases and phosphatases maintain the balance of protein phosphorylation, which is crucial for normal cellular activities [2]. SHP2, encoded by the PTPN11 gene, is a non-receptor protein tyrosine phosphatase (PTP) containing two SH2 domains and a PTP catalytic domain. It is the only confirmed proto-oncogene the PTP family [3]. Activating mutations or overexpression of SHP2 can lead to various solid tumors, such as leukemia, lung adenocarcinoma, colon cancer, and melanoma [[4], [5], [6]]. Abnormally activated SHP2 synergizes with receptor tyrosine kinases (RTKs) and KRAS signaling to promote tumor cell growth [7]. SHP2 also participates in JAK/STAT, PI3K/AKT, and PD-1/PD-L1 pathways through its interaction with the RAS/MAPK pathway [8,9]. Moreover, SHP2 acts as a downstream sensor of PD-1 signaling and can suppress T cell immune responses against tumors [10]. Therefore, SHP2 has become an attractive target, and the development of selective SHP2 inhibitors is of great significance.

SHP2 had been considered "undruggable" for decades. Early inhibitors, such as PHPS1 [11] and GS-493 [12], bind to the conserved PTP catalytic domain and block substrate access. However, due to the high conservation of this domain among PTPs, these inhibitors also affect other phosphatases required for normal physiology, leading to poor selectivity and clinical limitations [13].

In recent years, allosteric inhibitors of SHP2 have attracted increasing attention. In its inactive state, SHP2 is autoinhibited by interaction between its N-SH2 and PTP domains. Upon stimulation, binding of the SH2 domains to phosphotyrosine sites induces conformational changes that expose the catalytic pocket [14,15]. In 2016, the first allosteric inhibitor, SHP099, was reported, marking a major shift away from targeting the PTP domain [16]. Since then, several allosteric inhibitors, including TNO155 (Novartis) [17], JAB-3068 (Jacobio), and RMC-4630 (Sanofi) [18], have entered phase I/II clinical trials.

**Fig. 1:**
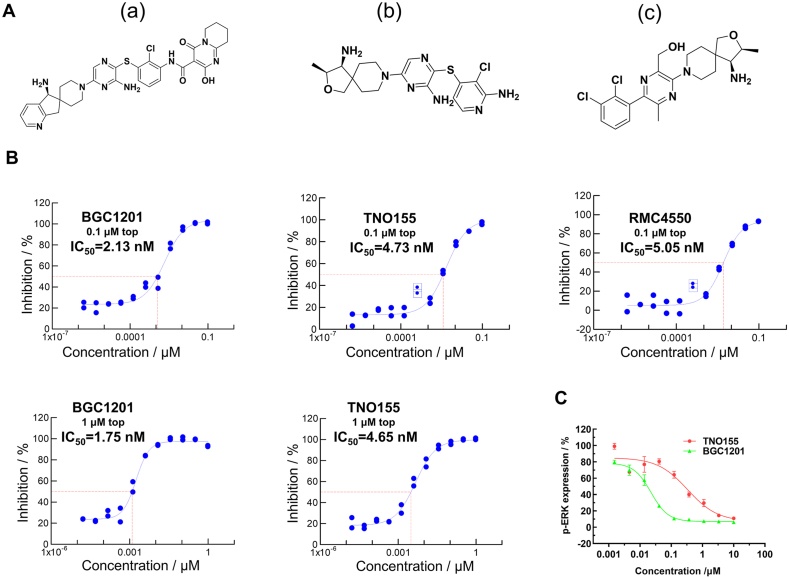
Structure and *in vitro* inhibitory activity of BGC1201. A: Structures of BGC1201 (a), TNO155 (b), and RMC4550 (c). B: Inhibitory activity of BGC1201, TNO155, and RMC4550 against SHP2-WT phosphatase. C: Effect of BGC1201 on p-ERK expression levels in NCI-H358 cells.

Despite these advances, existing SHP2 inhibitors still face limitations such as insufficient activity, a narrow safety window, and a high risk of hERG inhibition [19]. To explore potential strategies for improving SHP2 inhibitory activity and reducing hERG-related liability, we developed the novel clinical candidate BGC1201 using a fragment-based drug design (FBDD) strategy, which optimizes the structure of common SHP2 inhibitors that cause hERG inhibition (Fig. 1A). This study preliminarily evaluated the antitumor activity of BGC1201 at the enzymatic, cellular, and animal levels, thereby providing a basis for its further development.

## 2 Materials and methods

### 2.1 Materials and chemicals

BGC1201 and TNO155 were synthesized by BrightGene Bio-Medical (Suzhou) Co., Ltd., with purities of 99.4% and 98.8% respectively. RMC4550 was purchased from Selleck (purity 99.0%). Fetal bovine serum (FBS), culture media, Trypsin-EDTA were purchased from Gibco. Phosphate buffer solution was purchased from HyClone, and Matrigel was obtained from Corning. Trypsin, methanol, and acetonitrile were purchased from Honeywell. Trifluoroacetic acid (TFA), dimethyl sulfoxide (DMSO), and Tween 80 were purchased from Sinopharm Chemical Reagents Co., Ltd. All reagents used were of analytical grade or higher.

### 2.2 hERG potassium channel inhibition assay

To preliminarily evaluate the hERG-related potential cardiac safety liability of BGC1201 and compare it with TNO155, the inhibitory effects of both compounds on hERG potassium channel currents were assessed using CHO-hERG cells and a QPatch automated patch-clamp system. CHO-hERG cells were cultured to 60%–80% confluence, washed with PBS, detached with Detachin, neutralized with culture medium, centrifuged, and resuspended at a density of 5 × 10^6^ cells/mL for subsequent electrophysiological recording.

hERG currents were recorded using the QPatch automated patch-clamp system. After formation of the whole-cell recording configuration, cells were held at −80 mV. hERG tail currents were elicited by a depolarizing pulse to +40 mV followed by repolarization to −50 mV. The voltage protocol was repeated every 15 s. After a stable baseline was obtained, BGC1201 or TNO155 was applied sequentially from low to high concentrations at 0.16, 0.49, 1.48, 4.44, 13.33, and 40 μM. Each concentration was applied for 2.5 min, and at least three cells were recorded for each concentration. IC_50_ values were calculated based on the concentration-dependent inhibition of hERG current.

### 2.3 Assay of inhibitory activity against SHP2-WT phosphatase

The SHP2-WT phosphatase activity was measured according to a previously reported method [20]. SHP2-WT phosphatase and the test compound were added to the assay plate and centrifuged. After addition of the activating peptide solution, the plate was centrifuged again and incubated at 23 °C for 30 min. DiFMUP solution was then added, followed by centrifugation and further incubation at 23 °C for 30 min. The fluorescence signal was recorded using an EnVision plate reader, and the inhibition rate was calculated based on the Emission RFU signal at 450 nm.

### 2.4 Determination of p-ERK expression in NCI-H358 cells

NCI-H358 cells in the logarithmic growth phase were seeded into 96-well plates. A total of 200 μL of cell suspension was added to each well at a density of 2 × 10^4^ cells per well. Blank control wells were filled with culture medium without cells, and the plates were incubated overnight. After incubation, the medium in each well was removed and replaced with 45 μL of serum-free medium. Then, 5 μL of the compound (BGC1201, TNO155) was added and the cells were treated for 6 h. After treatment, the medium was removed, and 50 μL of freshly prepared lysis buffer was added. The plate was shaken at 350 rpm for 10 min. 10 μL of the lysate supernatant was transferred to an AlphaPlate-384 well plate containing 20 μL of lysis buffer, and then mixed for detection. Then, 15 μL of Acceptor Mix was added to the AlphaPlate-384 well, and the plate was incubated for 1 h in the dark. After that, 15 μL of Donor Mix was added in the dark and the plate was further incubated for 1 h. The signal was measured using an EnVision 2104 plate reader.

### 2.5 Antiproliferative activity in tumor cells

All cell were cultured under normal conditions and were passaged at least twice before seeding. Capan-1 and HUVEC cells were obtained from the American Type Culture Collection (ATCC), SK-LU-1 cells were obtained from Peking Union Medical College (CAMS), KYSE-520 cells were obtained from Cobioer, NCI-H441 and ECA109 cells were obtained from Wuhan University Cell Bank (CCTCC), and the others were obtained from the Cell Bank of Chinese Academy of Sciences (CAS).

On the day of the experiment, 100 μLof cell suspension was added to each well of a 96-well plate. 100 μL of BGC1201 or TNO155 working solutions with serial concentrations (0, 0.610, 2.44, 9.77, 39.1, 156, 630, 2500, 10000 nM) was added, with three duplicate wells in each group. After 5 days of drug treatment, the fluorescence intensity of each well was measured using the Alamar Blue assay, and the results were recorded.

### 2.6 Antitumor activity in vivo

Female BALB/c nude mice (SPF grade, a total of 165 mice) were purchased from Vital River Laboratory Animal Technology Co., Ltd. (animal certificate No. 20210722Abzz0619000181). All animal experiments were approved by the Institutional Animal Care and Use Committee of Suzhou Sushin New Drug Development Co., Ltd. (Approval No. 3D_ACUC2021009) and conducted in accordance with the regulations of the Association for Assessment and Accreditation of Laboratory Animal Care (AAALAC).

NCI-H358, MIA PaCa-2, or KYSE-520 cells in the logarithmic growth phase were collected and subcutaneously inoculated into the right axillary region of nude mice. The MIA PaCa-2 xenograft experiment was performed in a separate experimental batch from the NCI-H358 and KYSE-520 xenograft experiments. When the tumor volume reached 104 - 174 mm^3^, the animals were randomly assigned into five groups according to tumor volume, with eight mice in each group (n = 8). The solvent control and positive control group were respectively administered ultra-pure water and 30 mg kg^−1^ TNO155 (QD × 21) by gavage. The low, medium and high dose groups were respectively administered BGC1201 at 0.3, 1 and 3 mg kg^−1^ (KYSE-520 model, QD × 21), 0.3, 3 and 30 mg kg^−1^ (MIAPaCa-2 model, QD × 21), or 3, 10 and 30 mg kg^−1^ (NCI-H358 model, QD × 21), with a dosage volume of 10 mL kg^−1^. The model-specific dose ranges were selected based on preliminary dose-ranging observations and the pharmacodynamic responses of each xenograft model, aiming to cover a low-to-high efficacy range and to evaluate the dose-dependent antitumor activity of BGC1201 within each model. The experiment was terminated after 21 days of treatment. On day 22, the tumor size was measured and body weight was recorded. The animals were euthanized, and the tumor tissues were removed for photography and weighing.

### 2.7 Western blot analysis

The tumor tissues from nude mice treated with BGC1201 for 0, 1, 2, 4, 8, and 24 h were assigned to Group A, B, C, D, E, and F. A portion of the tissue was cut on ice, and lysed in cell lysis buffer containing protease inhibitors and phosphatase inhibitors, followed by centrifugation. The protein concentration in the supernatant was measured using a BCA protein assay kit. Based on the quantitative results, protein samples for loading were prepared, and the protein concentration of all samples was adjusted to 2 μg μL^−1^. The protein samples were separated by SDS-PAGE and transferred to membranes. The membrane was then incubated overnight at 4 °C with the primary antibodies against β-Actin, phospho-p44/42 MAPK (Erk1/2), and p44/42 MAPK (Erk1/2). The membranes were then incubated with the secondary antibody for 2 h at room temperature. Finally, the protein bands were visualized using a Western blot assay kit.

### 2.8 Pharmacokinetic evaluation

A total of 24 SD rats were randomly assigned to four groups, with six rats in each group and an equal number of males and females. The animals received a single intravenous dose of BGC1201 at 0.5 mg/kg or a single oral dose at 2, 6, or 20 mg/kg. Blood samples were collected pre-dose and at 0.083, 0.25, 0.5, 1, 2, 4, 8, 12, and 24 h after administration. Blood samples were anticoagulated with EDTA-K_2_ and centrifuged to obtain plasma.

The plasma concentrations of BGC1201 were determined using a validated LC-MS/MS method. Pharmacokinetic parameters were calculated using non-compartmental analysis (NCA) with WinNonlin 6.4. To evaluate potential sex-related differences in systemic exposure within each group, C_max_, AUC_0-t_, and AUC_0-∞_ were natural log-transformed and analyzed using a linear mixed-effects model.

### 2.9 Metabolite analysis

Plasma samples from the high-dose group of the SD rat pharmacokinetic study, including pre-dose and post-dose samples collected at 0.25, 0.5, 1, 2, 4, 6, 8, 12, and 24 h, were pooled by sex using the AUC pooling method. Urine and feces samples from the rat excretion study, collected at 0-4, 4-8, 8-12, 12-24, 24-48, 48-72, and 72-96 h, and bile samples collected at 0-4, 4-8, 8-12, 12-24, 24-36, and 36-48 h, were also pooled separately by sex.

BGC1201 and its potential metabolites were extracted from plasma, urine, feces, and bile samples using protein precipitation. The extracted samples were separated by high-performance liquid chromatography and analyzed by LC-MS/MS. Potential metabolites were initially screened by comparing the full-scan MS chromatograms of post-dose samples with those of pre-dose blank samples to identify major differential peaks. These potential metabolites were then subjected to MS/MS analysis. Their structures were proposed and characterized based on the structure of the parent compound, precursor ions, product ions, and characteristic fragmentation patterns.

### 2.10 Statistical analysis

Statistical analysis was performed using GraphPad Prism 9. Comparisons among multiple groups were conducted using one-way ANOVA followed by Tukey's multiple comparisons test for pairwise comparisons. Unless otherwise specified, all data are presented as the mean ± standard deviation (SD). A *P* value < 0.05 was considered statistically significant.

## 3 Results

### 3.1 Evaluation of hERG liability of BGC1201

BGC1201 showed weak inhibition of hERG current, with an IC_50_ value > 40 μM, while TNO155 showed an hERG IC_50_ value > 25 μM. These results suggest that BGC1201 has a low hERG-related liability in this preliminary in vitro assay.

### 3.2 BGC1201 potently inhibits SHP2-WT phosphatase

The IC_50_ values for the inhibition of SHP2-WT phosphatase by BGC1201, RMC4550, and TNO155 were 2.47 nM, 5.47 nM, and 5.41 nM respectively (Fig. 1B). These results indicate that BGC1201 exhibited stronger inhibitory activity against SHP2-WT phosphatase than TNO155 and RMC4550.

### 3.3 BGC1201 suppresses p-ERK expression in NCI-H358 cells

The changes in p-ERK expression after 6 h of treatment with BGC1201 in NCI-H358 cells are shown in Fig. 1C, with TNO155 used as the positive control. BGC1201 significantly inhibited the expression of p-ERK in the NCI-H358 cell line, with an IC_50_ of 0.0173 μM. The IC_50_ of TNO155 for inhibiting the expression of p-ERK was 0.2662 μM.

### 3.4 BGC1201 exhibits broad antiproliferative activity across 12 tumor cell lines

**Fig. 2:**
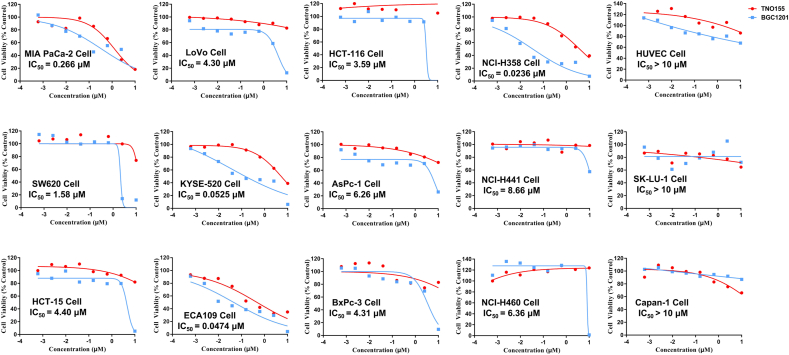
Curve fitting results of BGC1201 and TNO155 in 14 tumor cell lines and HUVEC cells.

BGC1201 significantly inhibited the proliferation of MIA PaCa-2, LoVo, HCT-116, NCI-H358, SW620, KYSE-520, AsPC-1, NCI-H441, HCT-15, ECA109, NCI-H460, BxPC-3 tumor cells, with IC_50_ values ranging from 0.0236 to 8.66 μM (Fig. 2). It had no significant inhibitory effect on SK-LU-1 and Capan-1 tumor cells and HUVEC cells, with IC_50_ values all greater than 10 μM. The IC_50_ value of TNO155 in MIA PaCa-2, NCI-H358, KYSE-520, SW620, ECA109 tumor cells was between 0.967 and 9.15 μM. It had no significant inhibitory effect on LoVo, Capan-1, HCT-116, SK-LU-1, AsPC-1, NCI-H441, HCT-15, NCI-H460, BxPC-3 and HUVEC cells, with IC_50_ values all greater than 10 μM. BGC1201 showed significant antiproliferative activity against multiple tumor cells and had no inhibitory effect on normal human umbilical vein endothelial cells. These results indicate that BGC1201 showed significant antiproliferative activity against tumor cells, together with a favorable safety profile in vitro.

### 3.5 BGC1201 demonstrates superior in vivo antitumor efficacy in three xenograft models

**Fig. 3:**
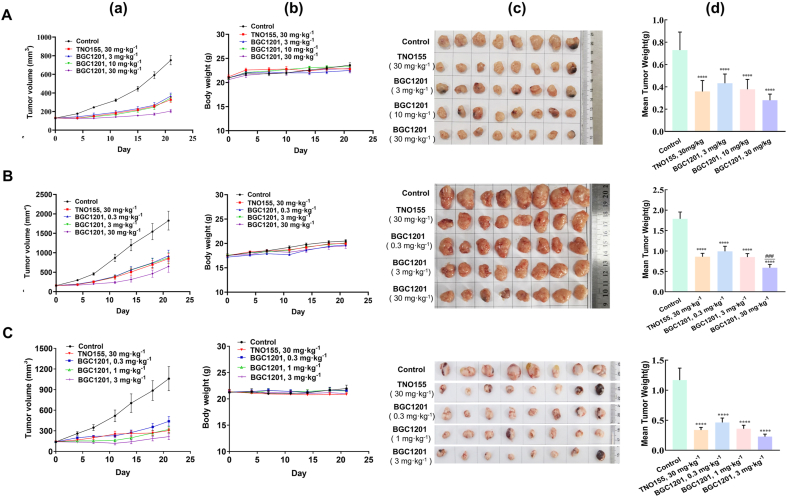
Effects of BGC1201 in nude mice xenograft models. A: Human lung cancer NCI-H358 xenograft model; B: Human pancreatic cancer MIAPaCa-2 xenograft model; C: Human esophageal cancer KYSE-520 xenograft model. (a) Tumor volume changes; (b) Body weight changes; (c) Tumor photographs; (d) Tumor weight histograms. n = 8, *∗P* < 0.05, *∗∗P* < 0.01, *∗∗∗P* < 0.001, *∗∗∗∗P* < 0.0001 *vs* Control; ^*###*^*P* < 0.001 *vs* TNO155 30 mg kg^−1^.

The antitumor activity of BGC1201 was evaluated in different nude mouse xenograft models, including the NCI-H358, MIA PaCa-2, and KYSE-520 models (Fig. 3). In the human lung cancer NCI-H358 xenograft model, the tumor volume of each group was approximately 130 mm^3^ on the first day. On the 22nd day, the tumor volume of the solvent control group was approximately 752 mm^3^. The tumor volumes of the low, medium and high-dose groups of BGC1201 administration were 367, 347 and 205 mm^3^ respectively, and the tumor growth inhibition (TGI) rates were 62%, 65% and 88%. The TGI rate in the positive control group treated with TNO155 was 68%. In the human pancreatic cancer MIAPaCa-2 xenograft model, the tumor volume of each group was approximately 158 mm^3^ on the first day. On the 22nd day, the tumor volume of the solvent control group was approximately 1827 mm^3^. The tumor volumes of the low, medium and high-dose groups of BGC1201 administration were 922, 873 and 649 mm^3^ respectively, and the TGI rates were 54%, 57% and 71%. The TGI rate in the positive control group was 59%. In the human esophageal cancer KYSE-520 xenograft model, the tumor volume of each group was approximately 150 mm^3^ on the first day. On the 22nd day, the tumor volume of the solvent control group was approximately 1059 mm^3^. The tumor volumes of the low, medium and high-dose groups of BGC1201 administration were 439, 321 and 216 mm^3^ respectively, and the TGI rates were 67%, 80% and 92%. The TGI rate in the positive control group was 82%. These results demonstrate that BGC1201 markedly inhibited the growth of human lung cancer NCI-H358, human pancreatic cancer MIA PaCa-2, and human esophageal cancer KYSE-520 xenografts in nude mice. Moreover, the antitumor effect of BGC1201 showed a clear dose-dependent trend.

### 3.6 BGC1201 reduces p-ERK expression in tumor tissues in a time-dependent manner

**Fig. 4:**
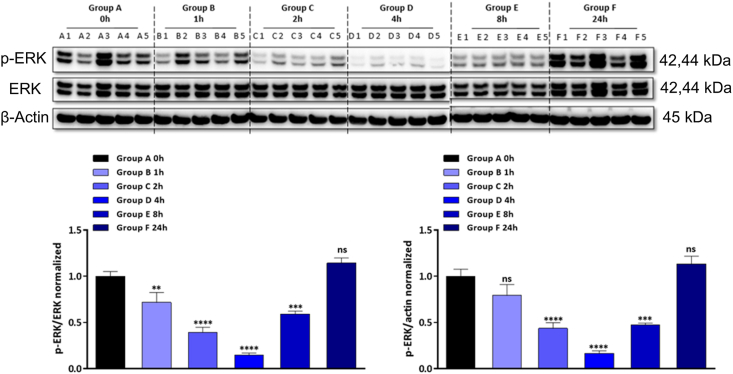
Western blot analysis of p-ERK protein expression in NCI-H358 tumor tissues. n = 5, *∗∗P* < 0.01, *∗∗∗P* < 0.001, *∗∗∗∗P* < 0.0001 *vs* Group A 0h.

The effects of BGC1201 on the expression of p-ERK and ERK were further investigated by Western blot analysis. The expression level of p-ERK in NCI-H358 tumor tissues after BGC1201 treatment was analyzed (Fig. 4). The results indicated that compared with the control group, the level of p-ERK in tumor tissues significantly decreased after treatment with BGC1201 for 1, 2, and 4 h, and it showed a certain time-dependent trend. After 8 h, the level of p-ERK gradually recovered. In contrast, BGC1201 did not affect the expression of ERK in tumor tissues.

### 3.7 Pharmacokinetic study

**Fig. 5:**
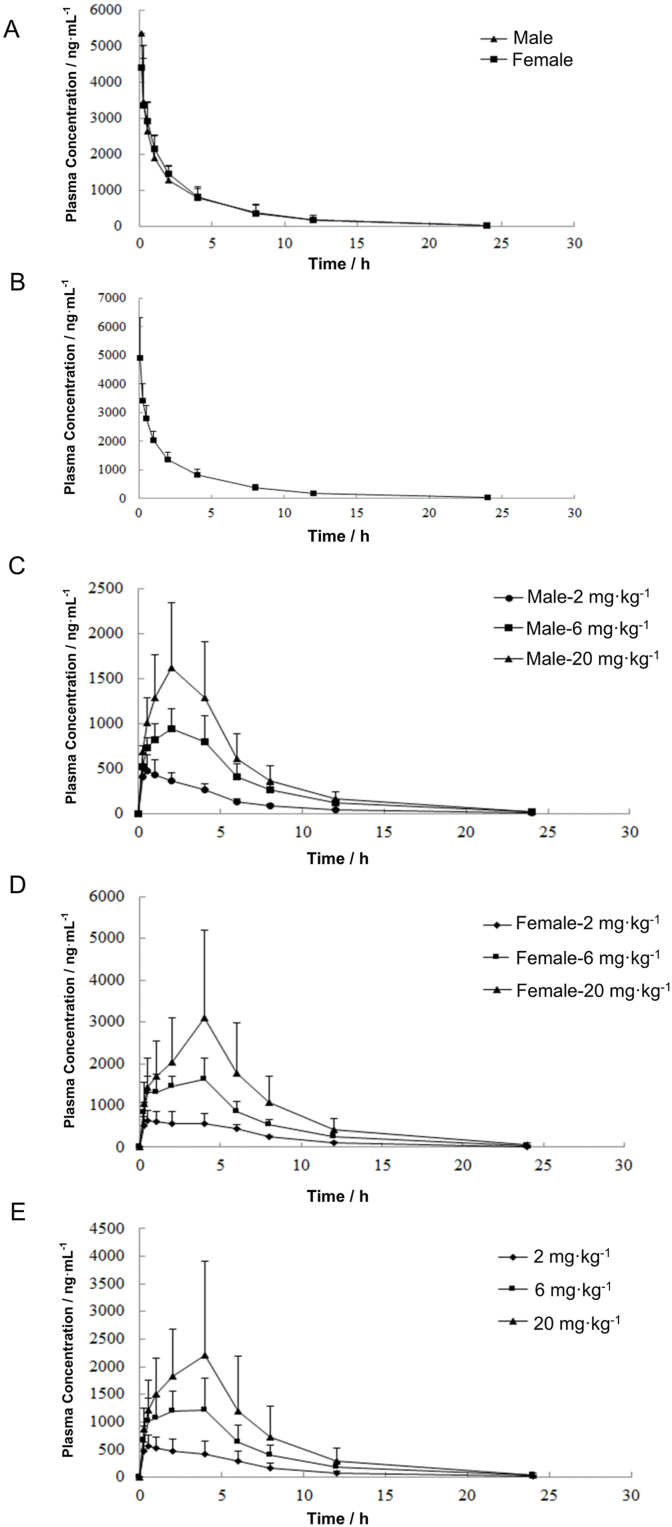
Plasma concentration–time curves of BGC1201 in SD rats after single intravenous or oral administration. (A) Male and female rats after intravenous administration; (B) Pooled rats after intravenous administration; (C) Male rats after oral administration; (D) Female rats after oral administration; (E) Pooled rats after oral administration.

To further evaluate the drug-like properties of BGC1201, its pharmacokinetic profile was investigated in SD rats after a single intravenous or oral administration (Table 1). Following a single intravenous dose of 0.5 mg/kg BGC1201, the mean elimination half-life (t_1/2_) was 4.29 h, and the AUC_0-t_ and AUC_0-∞_ values were 11741 and 11924 ng h/mL, respectively. The mean plasma clearance (CL), steady-state volume of distribution (Vss), and MRT_0-∞_ were 0.732 mL/min/kg, 0.195 L/kg, and 4.54 h, respectively, indicating low plasma clearance and relatively high systemic exposure in rats (Fig. 5A and B).

**Table 1:** Pharmacokinetic parameters in rats after intravenous or oral administration.

Group	Dose (mg/kg)	t_1/2_ (h)	T_max_ (h)	C_max_ (ng/mL)	AUC_0-t_ (ng·h/mL)	AUC_0-∞_ (ng·h/mL)	MRT_0-∞_ (h)	CL (mL/min/kg)	V_SS_ (L/kg)	F (%)
Intravenous group	0.5	4.29	/	/	11741	11924	4.54	0.732	0.195	/
Oral low-dose group	2	5.57	1.67	567	4064	4218	6.66	/	/	8.84
Oral medium-dose group	6	4.39	2.50	1380	9748	9940	6.01	/	/	6.95
Oral high-dose group	20	4.04	3.00	2360	16058	16294	5.78	/	/	3.42

After single oral administration of BGC1201 at 2, 6, and 20 mg/kg, BGC1201 was rapidly absorbed, with mean T_max_ values of 1.67, 2.50, and 3.00 h, respectively. The mean t_1/2_ values were 5.57, 4.39, and 4.04 h. As the oral dose increased, both C_max_ and AUC_0-t_ increased, with C_max_ values of 567, 1380, and 2360 ng/mL and AUC_0-t_ values of 4064, 9748, and 16058 ng h/mL, respectively. The mean oral bioavailability values were 8.84%, 6.95%, and 3.42% in the 2, 6, and 20 mg/kg groups, suggesting that BGC1201 achieved measurable systemic exposure after oral administration, although its absolute oral bioavailability remains to be further improved (Fig. 5C–E).

The dose-exposure relationship and potential sex difference were further analyzed after single oral administration. Overall, C_max_, AUC_0-t_, and AUC_0-∞_ increased in a dose-related manner when the dose was increased from 2 mg/kg to 6 and 20 mg/kg; however, the increase in systemic exposure was less than dose proportional. The mean exposure ratios between male and female rats were generally within a comparable range, with ratios of 1.37–1.92 for C_max_, 1.87–2.12 for AUC_0-t_, and 1.86–2.11 for AUC_0-∞_. Although the sex ratios of AUC_0-t_ and AUC_0-∞_ in the high-dose group were slightly greater than 2, independent-sample t-tests showed no statistically significant difference within this group (p > 0.05), indicating no apparent sex-related difference in systemic exposure (Table 2).

**Table 2:** Relationship between pharmacokinetic parameters and dose after oral administration in male and female rats.

Parameters	Sex	2 mg/kg	6 mg/kg	20 mg/kg	6 mg/kg vs 2 mg/kg	20 mg/kg vs 2 mg/kg
C_max_ (ng/mL)	Overall	567	1380	2360	2.43	4.16
AUC_0-t_ (ng·h/mL)		4064	9748	16058	2.40	3.95
AUC_0-∞_ (ng·h/mL)		4218	9940	16294	2.36	3.86
C_max_ (ng/mL)	Male	478	943	1620	1.97	3.39
AUC_0-t_ (ng·h/mL)		2723	6805	10310	2.50	3.79
AUC_0-∞_ (ng·h/mL)		2888	6951	10482	2.41	3.63
C_max_ (ng/mL)	Female	656	1810	3100	2.76	4.73
AUC_0-t_ (ng·h/mL)		5405	12692	21807	2.35	4.03
AUC_0-∞_ (ng·h/mL)		5549	12929	22106	2.33	3.98

### 3.8 Metabolite profiling of BGC1201 in rats

**Fig. 6:**
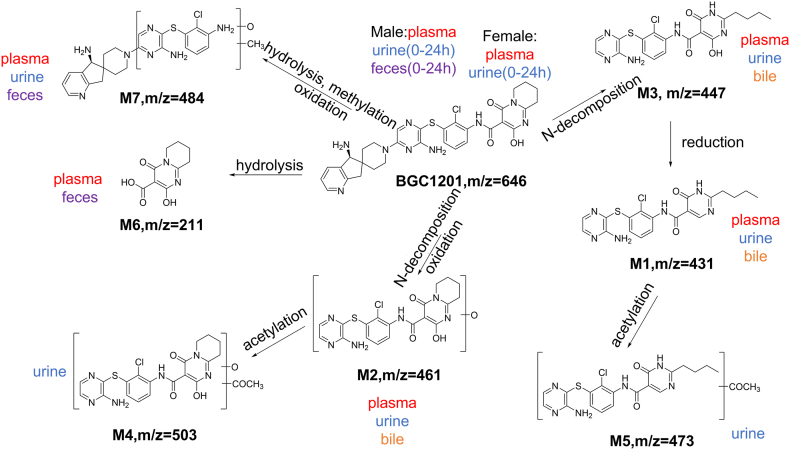
Major metabolites and proposed metabolic pathways of BGC1201 in rats.

To further characterize the metabolic profile of BGC1201 in rats, LC-MS/MS analysis was performed on plasma, urine, feces, and bile samples collected from SD rats. A total of seven major putative metabolites, designated M1–M7, were detected in rat samples (Table 3). Based on the precursor ions, product ions, and fragmentation patterns of the parent compound and its metabolites, the proposed metabolic pathways of BGC1201 mainly involved N-decomposition/dealkylation, hydrolysis, oxidation, reduction, methylation, and acetylation conjugation. The major metabolites and proposed metabolic pathways of BGC1201 in rats are shown in Fig. 6. The parent compound BGC1201 was detected in plasma, urine, and feces within 0–24 h after administration, suggesting that BGC1201 maintained measurable systemic exposure and could be eliminated through renal and gastrointestinal-related routes. Regarding metabolite distribution, M1, M2, and M3 were detected in plasma, urine, and bile, suggesting that these metabolites may be eliminated through both renal and biliary pathways. M4 and M5 were mainly detected in urine, indicating that they may be primarily excreted via the kidney after acetylation conjugation. M6 was mainly detected in feces, suggesting that gastrointestinal or biliary-fecal elimination may contribute to its clearance. Overall, BGC1201 underwent multiple phase I and phase II metabolic reactions in rats and was eliminated through renal, hepatobiliary, and fecal routes.

**Table 3:** Summary of major metabolites of BGC1201 in rats.

Metabolite	Metabolic reaction	Mass shift	Molecular formula	*m*/*z*(+H)	Retention time (min)	Fragment ion
Parent	NA	NA	C_31_H_32_ClN_9_O_3_S	646	9.70	629,454,437
M1	Reduction	−215	C_19_H_19_ClN_6_O_2_S	431	4.33	255
M2	N-decomposition, oxidation	−185	C_19_H_17_ClN_6_O_4_S	461	4.49	285
M3	N-decomposition	−199	C_19_H_19_ClN_6_O_3_S	447	5.04	271
M4	Acetylation	−143	C_21_H_19_ClN_6_O_5_S	503	5.69	255
M5	Acetylation	−173	C_21_H_21_ClN_6_O_3_S	473	6.02	251
M6	Hydrolysis	−435	C_9_H_10_N_2_O_4_	211	6.28	193,165,151
M7	Hydrolysis, methylation, oxidation	−162	C_23_H_26_ClN_7_OS	484	9.22	453,286

## 4 Discussion

SHP2, the only proto-oncoprotein in the PTP family, can be aberrantly activated and drive various malignancies, including leukemia and lung adenocarcinoma. Traditional inhibitors, such as PHPS1, competitively bind to the PTP catalytic domain. However, due to the high conservation of this domain across the PTP family, these inhibitors suffer from poor selectivity and low bioavailability, which long led to SHP2 being regarded as an "undruggable" target. The recent breakthrough of allosteric inhibitors has fundamentally changed this situation. BGC1201, developed using a FBDD strategy, optimizes the structural moiety associated with hERG toxicity, positioning it as a promising allosteric SHP2 inhibitor. However, the selectivity of BGC1201 over other PTP family members remains to be further determined.

BGC1201 demonstrated excellent in vivo antitumor activity, with significant and dose-dependent inhibition of tumor growth in all three xenograft models. In the NCI-H358 lung cancer model, BGC1201 at 10 mg kg^−1^ achieved antitumor efficacy comparable to that of TNO155 at 30 mg kg^−1^, while BGC1201 at 30 mg kg^−1^ produced a higher TGI than TNO155 at the same dose under the present experimental conditions. In the MIA PaCa-2 pancreatic cancer model, BGC1201 at 3 mg kg^−1^ showed efficacy comparable to that of TNO155 at 30 mg kg^−1^, and again the high dose (30 mg kg^−1^) was significantly better than TNO155 at the same dose. In the KYSE-520 esophageal cancer model, BGC1201 at 1 mg kg^−1^ was comparable to TNO155 at 30 mg kg^−1^, and the TGI rate at 3 mg kg^−1^ was higher than that of TNO155 at 30 mg kg^−1^.

The in vivo activity of BGC1201 was further contextualized by comparison with reported antitumor data for other SHP2 inhibitors or related targeted agents. For example, Kai Tang et al. reported that TK-642 [21]at 50 mg kg^−1^ achieved a TGI of 83.69% in the KYSE-520 model, whereas BGC1201 at 3 mg kg^−1^ achieved a TGI of 91.9% in the same model. Pang et al. [22] developed compound 4, an SOS1 degrader, which at 30 mg kg^−1^ inhibited NCI-H358 tumor growth by 58.8% after 21 days; under identical conditions, BGC1201 at 30 mg kg^−1^ yielded 88% TGI. Cheng et al. reported the KRASG12C inhibitor compound 20a [23], which at 15 mg kg^−1^ suppressed NCI-H358 tumor growth by approximately 50%; in the same model, BGC1201 at 3 mg kg^−1^ already achieved 62% TGI. Bharate et al. described IIIM-290 [24], a cyclin-dependent kinase inhibitor that at 50 mg kg^−1^ produced a 50% TGI in the MIA PaCa-2 model after 25 days; BGC1201 at 3 mg kg^−1^ reached 54% TGI after 21 days. These comparisons provide supportive context for the potent in vivo antitumor activity of BGC1201, although direct superiority requires head-to-head studies under matched experimental conditions.

SHP2 is a key node in the ERK signaling pathway, where it positively regulates RAS activation, relieves inhibitory regulation, and participates in feedback control to maintain signaling homeostasis. Aberrant SHP2 function leads to sustained ERK activation, driving tumorigenesis. Therefore, targeting SHP2 effectively suppresses ERK signaling, which is particularly valuable in RAS-mutant or RTK-dependent tumors. In this study, BGC1201 inhibited SHP2-WT phosphatase with an IC_50_ of 2.47 nM, showing superior potency compared to previously reported allosteric inhibitors such as SHP099 (IC_50_ = 71 nM) [16] and TK-453 (IC_50_ = 23 nM) [20]. BGC1201 also markedly reduced p-ERK levels in NCI-H358 cells (IC_50_ = 17.3 nM). Western blot analysis of NCI-H358 tumor tissues revealed a time-dependent decrease in p-ERK following BGC1201 treatment, with no change in total ERK expression. These results collectively demonstrate that BGC1201 exerts its antitumor activity by inhibiting SHP2 phosphatase activity, thereby suppressing the SHP2-mediated activation of the ERK signaling pathway.

Pharmacokinetic evaluation further supported the drug-like potential of BGC1201. After a single intravenous dose of 0.5 mg/kg in SD rats, BGC1201 showed an elimination half-life of 4.29 h and a plasma clearance of 0.732 mL/min/kg, suggesting relatively slow systemic clearance in rats. Following single oral administration at 2, 6, and 20 mg/kg, BGC1201 was rapidly absorbed, with T_max_ values ranging from 1.67 to 3.00 h. As the oral dose increased, both C_max_ and AUC_0-t_ increased accordingly. However, the increase in systemic exposure was less than dose-proportional, particularly at the high dose. The oral bioavailability of BGC1201 ranged from 3.42% to 8.84%, indicating that although measurable oral exposure was achieved, its absolute bioavailability remained relatively low. This may be associated with incomplete gastrointestinal absorption, first-pass metabolism, or formulation-related factors. Notably, despite its limited oral bioavailability, BGC1201 exhibited significant antitumor activity after oral administration in multiple xenograft models. The systemic exposure achieved under the current dosing regimens was sufficient to support in vivo pharmacodynamic activity. Further formulation optimization or structural modification may help improve the oral exposure and bioavailability of BGC1201, while PK/PD studies will be important to clarify its exposure-response relationship.

## 5 Limitation

This study has several limitations. First, although BGC1201 showed weak hERG inhibition in the in vitro automated patch-clamp assay, this result is not sufficient to fully establish its cardiac safety, and further in vivo cardiovascular safety pharmacology studies are required. Second, although BGC1201 achieved measurable systemic exposure after oral administration and supported in vivo antitumor activity, its oral bioavailability remained relatively low, which requires further investigation in relation to membrane permeability, first-pass metabolism, or formulation-related factors. Third, the selectivity of BGC1201 against representative PTP family members, such as SHP1, PTP1B, and TCPTP, has not been systematically evaluated. Further PTP enzymatic profiling and cellular pathway validation using markers such as p-STAT3 and p-AKT are needed to clarify its target specificity. Finally, the comparison between BGC1201 and TNO155 was not based on a complete head-to-head dose-response or PK/PD study, and future work should establish the exposure-response relationship among plasma exposure, tumor drug concentration and tumor growth inhibition.

## 6 Conclusion

This study systematically evaluated the antitumor activity of the novel SHP2 inhibitor BGC1201. In vitro results demonstrated that BGC1201 showed weak inhibition of the hERG potassium channel, suggesting a low potential hERG-related cardiac safety liability. BGC1201 also significantly inhibited SHP2-WT phosphatase activity, reduced p-ERK expression in NCI-H358 cells, and potently suppressed the proliferation of 12 tumor cell lines. In vivo pharmacodynamic studies further confirmed that BGC1201 exhibited significant and dose-dependent antitumor activity. Pharmacokinetic studies further supported the drug-like evaluation of BGC1201. This study not only supports the development of SHP2 allosteric inhibitors but also provides BGC1201 as a promising clinical candidate. Further investigations into the safety and efficacy of BGC1201 are warranted to facilitate its rational clinical application.

## Ethics approval

The mice used in this study were purchased Vital River Laboratory Animal Technology Co., Ltd. (certificate No. 20210722Abzz0619000181). All animal experiments were approved by the Institutional Animal Care and Use Committee of Suzhou Sushin New Drug Development Co., Ltd. (Approval No. 3D_ACUC2021009).

## Declaration of generative AI in scientific writing

Not applicable.

## Funding information

This work was supported by the 10.13039/501100012246Priority Academic Program Development of Jiangsu Higher Education Institutions (PAPD).

## CRediT authorship contribution statement

**Yuhuai Wang:** Data curation, Formal analysis, Investigation, Methodology, Writing – original draft. **Jiahui Xia:** Data curation, Formal analysis, Writing – original draft. **Haifeng Ding:** Conceptualization, Project administration, Writing – review & editing. **Duxin Li:** Supervision, Writing – review & editing.

## **Declaration of competing interest**

The authors declare that they have no known competing financial interests or personal relationships that could have appeared to influence the work reported in this paper.

## Data Availability

The data that support the findings of this study are available from the corresponding author upon reasonable request.
